# A Geometry-Based Beamforming Channel Model for UAV mmWave Communications

**DOI:** 10.3390/s20236957

**Published:** 2020-12-05

**Authors:** Kai Mao, Qiuming Zhu, Maozhong Song, Boyu Hua, Weizhi Zhong, Xijuan Ye

**Affiliations:** 1 The Key Laboratory of Dynamic Cognitive System of Electromagnetic Spectrum Space, College of Electronic and Information Engineering, Nanjing University of Aeronautics and Astronautics, Nanjing 211106, China; maokai@nuaa.edu.cn (K.M.); smz108@nuaa.edu.cn (M.S.); byhua@nuaa.edu.cn (B.H.); yexijuan@nuaa.edu.cn (X.Y.); 2 The Key Laboratory of Dynamic Cognitive System of Electromagnetic Spectrum Space, College of Astronautics, Nanjing University of Aeronautics and Astronautics, Nanjing 211106, China; zhongwz@nuaa.edu.cn

**Keywords:** UAV, beamforming channels, GBSM, mmWave communications, ray tracing

## Abstract

Considering the three-dimensional (3D) trajectory, 3D antenna array, and 3D beamforming of unmanned aerial vehicle (UAV), a novel non-stationary millimeter wave (mmWave) geometry-based stochastic model for UAV to vehicle communication channels is proposed. Based on the analysis results of measured and ray tracing simulation data of UAV mmWave communication links, the proposed parametric channel model is constructed by a line-of-sight path, a ground specular path, and two strongest single-bounce paths. Meanwhile, a new parameter computation method is also developed, which is divided into the deterministic (or geometry-based) part and the random (or empirical) part. The simulated power delay profile and power angle profile demonstrate that the statistical properties of proposed channel model are time-variant with respect to the scattering scenarios, positions and beam direction. Moreover, the simulation results of autocorrelation functions fit well with the theoretical ones as well as the measured ones.

## 1. Introduction

Unmanned aerial vehicles (UAVs) have been expected to be a promising platform to expand the communication range as flying relays in the fifth-generation (5G) and beyond fifth-generation (B5G) communication systems [1,2]. Meanwhile, the millimeter wave (mmWave) from 6 GHz to 100 GHz is also considered to be an important technique to provide high transmission rate and can be applied on UAVs for the small size of mmWave antennas [3,4]. However, the communication distance of UAVs is greatly limited due to the high path loss of mmWave propagation, and thus the three-dimensional (3D) beam-forming technology with antenna arrays has gathered more and more attention [4,5]. Beside these, UAV communication scenarios have some other special characteristics, i.e., 3D flying trajectory and 3D scattering space, which results in the existing channel models for traditional communication scenarios being not suitable any more [6,7]. Therefore, it is essential to deeply understand these new features and obtain a tailored channel model for the UAV mmWave communication system.

There has been growing interest in the UAV channel modeling, which can be classified into deterministic models [8,9] and stochastic models [10,11,12]. Among them, the geometry-based stochastic models (GBSMs) have been widely applied due to the good tradeoff between the generality, accuracy, and complexity [13,14,15,16]. In [13], a 3D non-stationary GBSM for the air-to-ground communication was proposed and both the air and ground terminals were moving. The authors in [14] proposed a novel UAV channel model with two cylinder scatterers around the transceiver. The authors in [15,16] upgraded the 3D GBSM by taking into account of the 3D flying trajectory and arbitrary velocity of UAV. However, all of above GBSMs only focused on the sub-mmWave frequency band and were inapplicable for the UAV mmWave channel.

So far, most of mmWave channel models were mainly aimed at the land mobile communication scenarios [17,18], e.g., tunnel [19,20], microcellular environment [21], and high-voltage substation [22], etc. A few mmWave channel models involving UAV scenarios can be addressed in [23,24,25,26]. In [23,24], the authors used ray tracing (RT) simulated data to develop UAV mmWave channel models and analyze the channel parameters, i.e., received power, path delay, angle, etc. It should be noted that the channel model was 2D in [23] and the ground terminal was fixed in [24]. Moreover, the RT-based channel modeling is generally time-consuming and has high complexity. In [25], a 3D non-stationary mmWave channel for UAV communication was proposed based on the geometry-based stochastic model (GBSM) method, but the flight velocity was constant and the receiving terminal was also fixed. Recently, the authors in [26] proposed a mmWave UAV channel model allowing 3D trajectories, but the rotation of 3D-shaped antenna array and the effect of beam-forming were not considered.

The GBSM is a great alternative to model the UAV mmWave beam channel, but we note that the existing GBSMs for UAV mmWave channel cannot completely cover the new features of UAV to vehicle (U2V) beam communications. Especially, the characteristic of 3D beam-forming is not included in the related literature, which would change the covering range of signal or the distribution of scatterers and furthermore affects the channel properties, i.e., path angles and power gains. This paper aims to fill these research gaps. The major contributions and novelties of this paper are summarized as follows:

(1) A 3D GBSM for U2V mmWave beam channel considering the 3D arbitrary trajectory, 3D antenna array, and 3D beam-forming of UAV is proposed. To achieve the tradeoff between generality, accuracy, and complexity, the model only takes into account the line-of-sight (LoS) path, ground specular (GS) path, and two strongest single-bounce (SB) paths.
(2) A hybrid computation method of channel parameters, i.e., geometry-based parameters and data-based parameters, for the proposed model is developed. The geometry-based parameters, e.g., the locations of terminals, the mean angles and delays of paths, are calculated by the time-variant but deterministic geometric relationships, and the data-based channel parameters, e.g., the angle offset and delay offset of the rays, the path powers, are generated randomly from the corresponding distribution fitted by RT simulation or measured data.
(3) Considering an urban U2V mmWave communication scenario, the channel parameters, i.e., path delays, received powers, and angles, are simulated and demonstrated. Moreover, the simulation results of the second order statistical properties, i.e., autocorrelation function (ACF) and Doppler power spectral density (DPSD), are also validated by theoretical and measured ones.

The rest paper is organized as follows. In Section 2, a 3D GBSM for U2V mmWave beam channel is proposed. Section 3 gives the computation method of geometry-based parameters and data-based parameters. The simulation and analytical results of the channel parameters and statistical properties are given in Section 4. Finally, conclusions are drawn in Section 5.

## 2. UAV mmWave Channel Model

**Figure 1:**
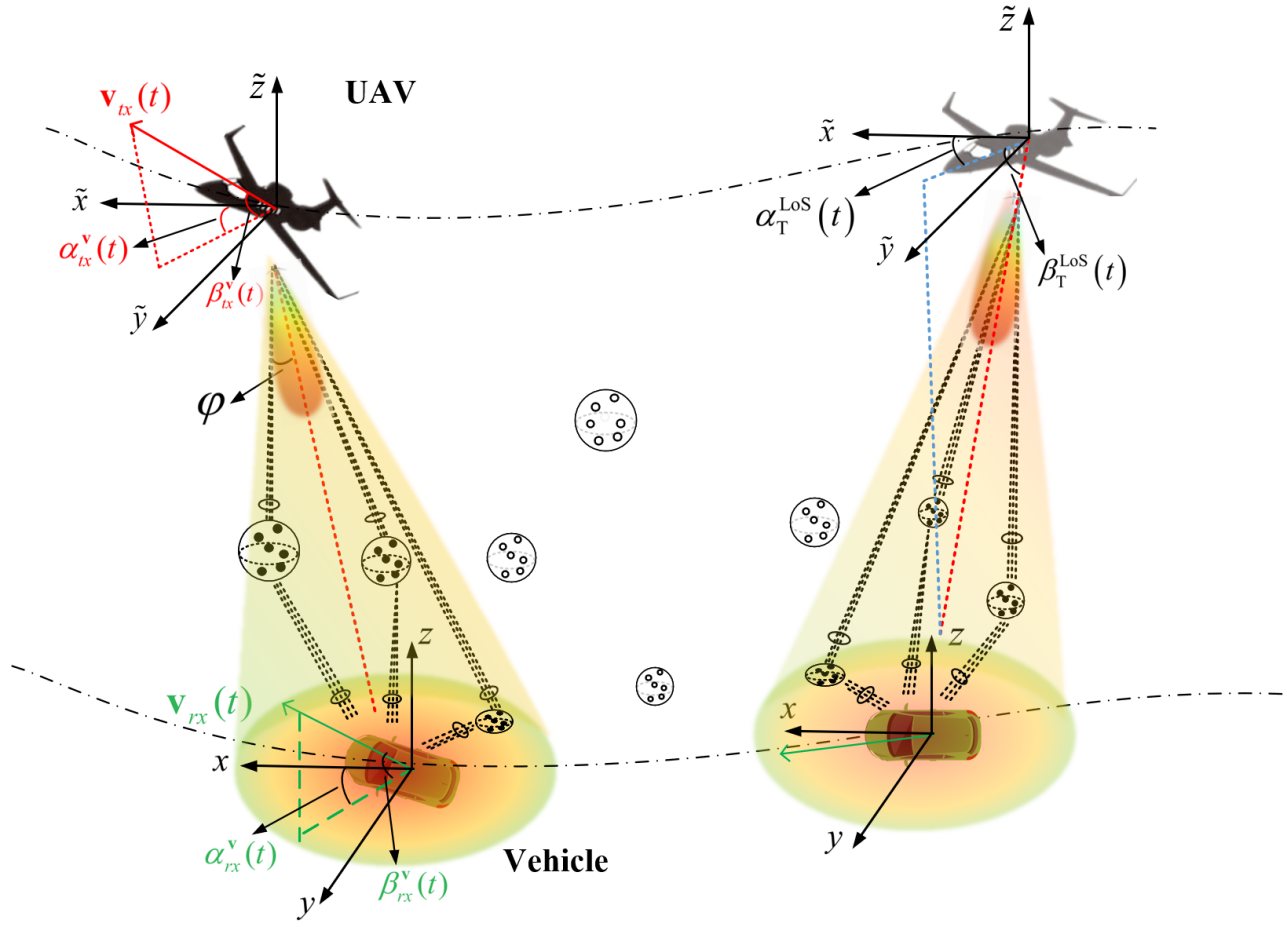
Typical U2V mmWave beam channel.

Let us consider a typical U2V communication scenario as shown in Figure 1, where the 3D beamforming is applied to compensate the high path loss. The vehicle is equipped with omnidirectional antennas. Within the beam, the gain coefficient from the antenna array varies with the angle offset between the beam center and different paths, which would affect the power gain of propagation channel. In Figure 1, two independent coordinate systems are denoted as the UAV coordinate system and vehicle coordinate system with their origins at the central of UAV and vehicle, respectively, and the arbitrary velocities of UAV or vehicle can be denoted as

(1) $$v_{T/R}\left(t\right)=v_{T/R}\left(t\right)\left[\begin{array}{c} \cos\alpha_{T/R}^{v}\left(t\right)\cos\beta_{T/R}^{v}\left(t\right)\\ \sin\alpha_{T/R}^{v}\left(t\right)\cos\beta_{T/R}^{v}\left(t\right)\\ \sin\beta_{T/R}^{v}\left(t\right) \end{array}\right]$$

where $v_{T/R}\left(t\right)$, $\alpha_{T/R}^{v}\left(t\right)$ and $\beta_{T/R}^{v}\left(t\right)$ are the magnitude, azimuth angle, and elevation angle of $v_{T/R}\left(t\right)$, respectively. It should be noted that ${\left(\cdot \right)}_{T/R}$ represents two equations for T and R, respectively.

**Figure 2:**
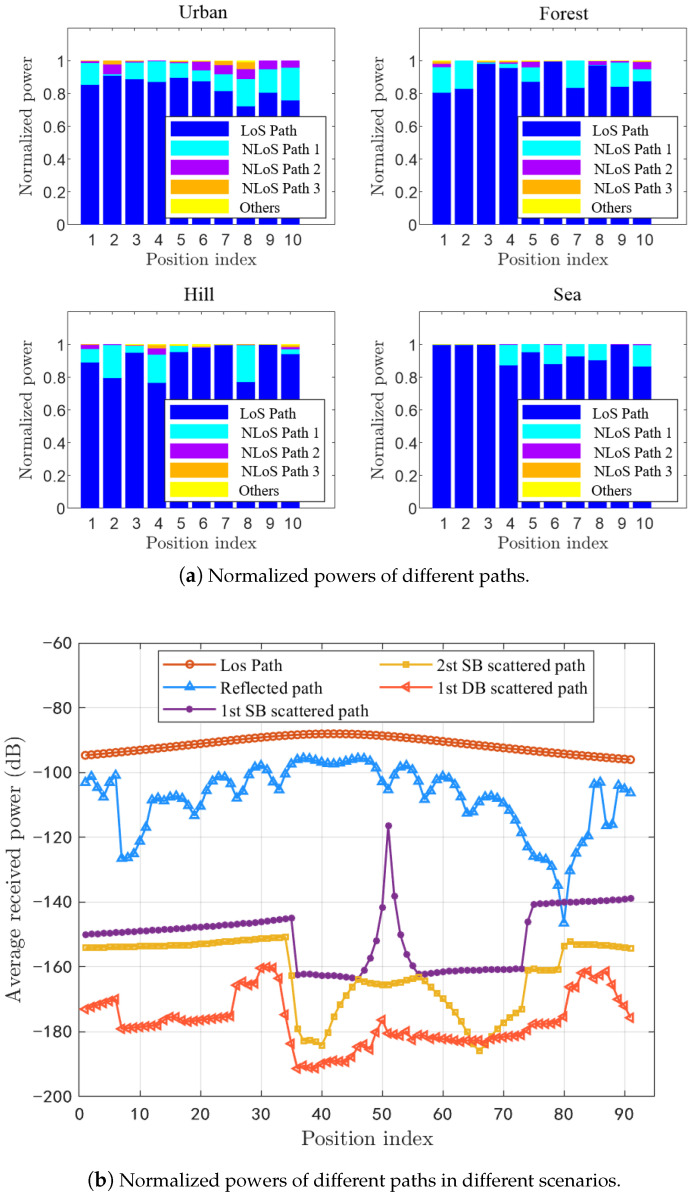
Analyzed results of path power for U2V mmWave channels.

The U2V beamforming propagation channel normally includes a LoS path and tremendous NLoS paths, i.e., GS path, SB paths, double-bounce (DB) paths, etc. To simplify the channel model, we have performed large amount of RT simulations and obtained massive raw data of mmWave U2V channels in 28 GHz under four scenarios, i.e., urban, forest, hill, and sea. It is assumed that the LoS path always exists during the simulation. As shown in Figure 2a, we find that the powers of LoS path and three strongest paths are at least 90% and 95% of the total one, respectively. Moreover, the summed power of four strongest paths is over 99% of the total one and thus the path number of channel model can be simplified into 4. Furthermore, Figure 2b gives more details of the received powers of different paths. In the figure, the UAV flies through several different trajectories under the urban scenario and the received powers are averaged. It can be seen that the power of the LoS path is normally 20 dB and 40 dB more than the GS path and the SB paths, respectively. Moreover, the power of the strongest DB path is lower than the ones of strongest SB path and second strongest SB path, and thus the four strongest paths normally represent the LoS path, GS path, 1st SB path, and 2st SB path.

Moreover, it is assumed that the LoS path includes one direct ray and each NLoS path includes several non-direct rays with similar delays. Therefore, in this paper the channel impulse response (CIR) between the *p*th UAV antenna and the *q*th vehicle antenna scaled by the K-factor *K* only includes the LoS path $h_{}^{\mathrm{LoS}}\left(t\right)$ and three strongest NLoS paths, i.e., $h_{}^{\mathrm{GS}}\left(\tau ,t\right)$, $h_{}^{SB_{1}}\left(\tau ,t\right)$, $h_{}^{SB_{2}}\left(\tau ,t\right)$ as

(2) $$\begin{array}{cc} h(\tau ,t) & =h_{}^{\mathrm{LoS}}\left(t\right)+\underset{ground\;specular}{\underset{︸}{h_{}^{\mathrm{GS}}\left(\tau ,t\right)}}+\underset{Single\;bounce}{\underset{︸}{h_{}^{SB_{1}}\left(\tau ,t\right)+h_{}^{SB_{2}}\left(\tau ,t\right)}}\\ \; & =\sqrt{\frac{K(t)}{K(t)+1}}A\left(\theta^{\mathrm{LoS}},\varphi^{\mathrm{LoS}}\right){\tilde{h}}_{}^{\mathrm{LoS}}\left(t\right)\\ \; & \;\;\;\;\;\;\;+\sqrt{\frac{1}{K(t)+1}}\sum_{j=1}^{3}\sum_{m=1}^{M}A\left(\theta_{m}^{j},\varphi_{m}^{j}\right)\sqrt{P_{m}^{j}\left(t\right)}{\tilde{h}}_{m}^{j}\left(t\right)\delta \left(t-\tau_{m}^{j}\right) \end{array}$$

where *M* is the ray number of each NLoS path, $j\in \left\{1,2,3\right\}$ represents the GS path, SB1 path and SB2 path, respectively. In (2), $A(\theta^{\mathrm{LoS}},\varphi^{\mathrm{LoS}})$ and $A(\theta_{m}^{j},\varphi_{m}^{j})$ are the gain coefficient of LoS path and NLoS paths within the beam, respectively, $P_{m}^{j}\left(t\right)$ and $\tau_{m}^{j}\left(t\right)$ are the power and delay of the *m*th ray within the *j*th NLoS path, respectively. Moreover, ${\tilde{h}}_{m}^{j}\left(t\right)$ denotes the channel coefficient of *m*th ray and can be modeled as

(3) $$\begin{array}{cc} {\tilde{h}}_{m}^{j}\left(t\right) & =\exp\left(j\Phi_{I}\right)\exp\left(j2\pi \frac{r_{R,m}^{j}\left(t\right)\cdot R_{R}^{}\left(t\right)\cdot d_{R}\left(t\right)}{\lambda }\right)\\ & \;\;\;\cdot \exp\left(j2\pi \frac{r_{T,m}^{j}\left(t\right)\cdot R_{T}\left(t\right)\cdot d_{T}\left(t\right)}{\lambda }\right)\cdot \exp\left(j2\pi \int_{t_{0}}^{t}f_{m}^{j}\left(t^{\prime }\right)dt^{\prime }\right) \end{array}$$

where $\Phi_{I}$ represents the random initial phase distributed uniformly over [0,2π), λ is the wavelength, $t_{0}$ is the initial time instant, and $d_{T/R}\left(t\right)$ denotes the location vectors of UAV transmitting antenna (or vehicle receiving antenna) in their own coordinate systems and can be described as

(4) $$d_{T/R}\left(t\right)=\left[\begin{array}{c} d_{T/R}^{x}\left(t\right)\\ d_{T/R}^{y}\left(t\right)\\ d_{T/R}^{z}\left(t\right) \end{array}\right]$$

where $d_{T/R}^{x}\left(t\right)$, $d_{T/R}^{y}\left(t\right)$, and $d_{T/R}^{z}\left(t\right)$ represent the *x*, *y*, and *z* component of $d_{T/R}\left(t\right)$. In (3), $r_{T,m}^{j}\left(t\right)$ (or $r_{R,m}^{j}\left(t\right)$) and $f_{m}^{j}$ are the spherical unit vectors and Doppler frequency of the *m*th ray, respectively, and $r_{T,m}^{j}\left(t\right)$ (or $r_{R,m}^{j}\left(t\right)$) can be further expressed as

(5) $$r_{T/R,m}^{j}\left(t\right)=\left[\begin{array}{c} \cos\beta_{T/R,m}^{j}\left(t\right)\cos\alpha_{T/R,m}^{j}\left(t\right)\\ \cos\beta_{T/R,m}^{j}\left(t\right)\sin\alpha_{T/R,m}^{j}\left(t\right)\\ \sin\beta_{T/R,m}^{j}\left(t\right) \end{array}\right]$$

where $\alpha_{T/R,m}^{j}$ is the azimuth angle of departure (AAoD) or arrival (AAoA), $\beta_{T/R,m}^{j}$ is the elevation angle of departure (EAoD) or arrival (EAoA). During the movement of UAV and vehicle, the location of each antenna may change, and in this paper a rotation matrix $R_{T/R}\left(t\right)$ is introduced to take this factor into account as

(6) $$R_{T/R}\left(t\right)=\left[\begin{array}{ccc} \cos\alpha_{T/R}^{v}\left(t\right)\cos\beta_{T/R}^{v}\left(t\right) & -\sin\alpha_{T/R}^{v}\left(t\right) & -\cos\alpha_{T/R}^{v}\left(t\right)\sin\beta_{T/R}^{v}\left(t\right)\\ \sin\alpha_{\mathrm{tx}/\mathrm{rx}}^{v}\left(t\right)\cos\beta_{T/R}^{v}\left(t\right) & \cos\alpha_{T/R}^{v}\left(t\right) & -\sin\alpha_{T/R}^{v}\left(t\right)\sin\beta_{T/R}^{v}\left(t\right)\\ \sin\beta_{T/R}^{v}\left(t\right) & 0 & \cos\beta_{T/R}^{v}\left(t\right) \end{array}\right]$$

The LoS path between the UAV and vehicle can be viewed as a special case of NLoS path and the corresponding channel coefficient can be expressed as

(7) $$\begin{array}{c} {\tilde{h}}_{}^{\mathrm{LoS}}\left(t\right)=\exp\left(-j2\pi \frac{D_{\mathrm{LoS}}\left(t\right)}{\lambda }\right)\exp\left(j2\pi \frac{r_{R}^{\mathrm{LoS}}\left(t\right)\cdot R_{R}\left(t\right)\cdot d_{R}\left(t\right)}{\lambda }\right)\\ \;\;\;\;\;\;\;\;\;\;\;\cdot \exp\left(j2\pi \frac{r_{T}^{\mathrm{LoS}}\left(t\right)\cdot R_{T}\left(t\right)\cdot d_{T}\left(t\right)}{\lambda }\right)\exp\left(\frac{j2\pi }{\lambda }\int_{0}^{t}f_{}^{\mathrm{LoS}}\left(t^{\prime }\right))dt^{\prime }\right)\delta \left(t-\tau^{\mathrm{LoS}}\left(t\right)\right) \end{array}$$

where $D_{\mathrm{LoS}}\left(t\right)$ is the distance between the UAV and vehicle, $r_{T/R}^{\mathrm{LoS}}\left(t\right)$ and $f_{}^{\mathrm{LoS}}$ denote the spherical unit vectors and Doppler frequency of LoS path, respectively, and $r_{T/R}^{\mathrm{LoS}}\left(t\right)$ can be expressed by $\alpha_{T/R}^{\mathrm{LoS}}$ and $\beta_{T/R}^{\mathrm{LoS}}$ as

(8) $$\begin{array}{c} r_{T/R}^{\mathrm{LoS}}\left(t\right)=\left[\begin{array}{c} \cos\beta_{T/R}^{\mathrm{LoS}}\left(t\right)\cos\alpha_{T/R}^{\mathrm{LoS}}\left(t\right)\\ \cos\beta_{T/R}^{\mathrm{LoS}}\left(t\right)\sin\alpha_{T/R}^{\mathrm{LoS}}\left(t\right)\\ \sin\beta_{T/R}^{\mathrm{LoS}}\left(t\right) \end{array}\right]. \end{array}$$

## 3. Hybrid Computation Method of Channel Parameters

### 3.1. Geometry-Based Parameters

The deterministic part of channel parameters can be calculated according to the geometric relationships of communication scenario, i.e., locations and velocities of transceivers and scatterers. Since the UAV and vehicle move along with 3D arbitrary trajectories, the time-variant location vector of UAV (or vehicle) can be expressed as

(9) $$L_{T}\left(t\right)=L_{T}\left(t_{0}\right)+\int_{t_{0}}^{t}v_{T}\left(t\right)dt$$

(10) $$L_{R}\left(t\right)=L_{R}\left(t_{0}\right)+\int_{t_{0}}^{t}v_{R}\left(t\right)dt$$

where $L_{T/R}\left(t_{0}\right)$ denotes the initial location vector of UAV (or vehicle) at $t=t_{0}$. The distance vector between the UAV and vehicle $D_{\mathrm{LoS}}^{}\left(t\right)$ can be expressed as

(11) $$\begin{array}{cc} D_{\mathrm{LoS}}^{}\left(t\right) & =L_{T}^{}\left(t\right)-L_{R}^{}\left(t\right)\\ \; & =D_{\mathrm{LoS}}^{}\left(t_{0}\right)r_{T}^{\mathrm{LoS}}\left(t\right)+\int_{t_{0}}^{t}v_{T,R}\left(t\right)dt \end{array}$$

where $D_{\mathrm{LoS}}^{}\left(t_{0}\right)$ is the initial distance of LoS path and can be expressed as

(12) $$D_{\mathrm{LoS}}^{}\left(t_{0}\right)=∥L_{T}\left(t_{0}\right)-L_{R}\left(t_{0}\right)∥$$

And $v_{T,R}\left(t\right)$ is the relative velocity between the UAV and vehicle which can be expressed as

(13) $$v_{T,R}\left(t\right)=v_{T}\left(t\right)-v_{R}\left(t\right).$$

Similarly, the distance vector between the UAV (or vehicle) and *j*th scatterer $D_{T/R,j}^{}\left(t\right)$ can be expressed as

(14) $$\begin{array}{cc} D_{T/R,j}^{}\left(t\right) & =L_{T/R}^{}\left(t\right)-L_{j}^{}\left(t\right)\\ \; & =D_{T/R,j}^{}\left(t_{0};t\right)r_{T/R}^{j}\left(t\right)+\int_{t_{0}}^{t}v_{T/R}\left(t\right)dt \end{array}$$

where $D_{T/R,j}^{}\left(t_{0};t\right)$ is the initial distance between the UAV (or vehicle) and jth scatterer. In (14), $r_{T/R}^{j}\left(t\right)$ is the spherical unit vectors of each NLoS path, which can be expressed by the mean angles denoted by ${\bar{\alpha }}_{T/R}^{j}$ and ${\bar{\beta }}_{T/R}^{j}$ as

(15) $$r_{T/R}^{j}\left(t\right)=\left[\begin{array}{c} \cos{\bar{\beta }}_{T/R,m}^{j}\left(t\right)\cos{\bar{\alpha }}_{T/R,m}^{j}\left(t\right)\\ \cos{\bar{\beta }}_{T/R,m}^{j}\left(t\right)\sin{\bar{\alpha }}_{T/R,m}^{j}\left(t\right)\\ \sin{\bar{\beta }}_{T/R,m}^{j}\left(t\right) \end{array}\right]$$

Moreover, the scatterers in this paper are assumed to be static and the valid ones covered by the 3D beam will change along with the beam direction. To evolve the geometry parameter of distance, the initial distance should be refreshed when the distribution of the valid scatterers have changed. Therefore, the initial distance $D_{T/R,j}^{}\left(t_{0};t\right)$ can be rewritten as

(16) $$D_{T/R,j}^{}\left(t_{0};t\right)=D_{T/R,j}^{}\left(t_{0}+kT_{0}\right)W\left(t-kT_{0}\right),k=0,1,2...$$

where the window function W(·) is introduced and can be denoted as

(17) $$W\left(t\right)\overset{\Delta }{\;=}\left\{\begin{array}{c} 1{,}_{}^{}0\leq t\leq T_{0}\\ 0{,}_{}^{}\mathrm{otherwise} \end{array}\right.$$

where $T_{0}$ is the stationary interval of scatterers and it is related with the beam width and the velocity of both terminals. The distance between UAV and vehicle in the LoS scenario and the one between the UAV (or vehicle) and *j*th scatterer can be calculated respectively by

(18) $$D_{\mathrm{LoS}}^{}\left(t\right)=\sqrt{\begin{array}{c} {\left(D_{\mathrm{LoS}}^{}\left(t_{0}\right)\cos\left(\alpha_{T/R}^{\mathrm{LoS}}\left(t_{0}\right)\right)\cos\left(\beta_{T/R}^{\mathrm{LoS}}\left(t_{0}\right)\right)+\int_{t_{0}}^{t}\left(v_{T,R}\left(t\right)\right)\cdot \cos\left(\alpha_{T,R}^{v}\left(t\right)\right)\cdot \cos\left(\beta_{T,R}^{v}\left(t\right)\right)dt\right)}^{2}\\ +{\left(D_{\mathrm{LoS}}^{}\left(t_{0}\right)\cos\left(\beta_{T/R}^{\mathrm{LoS}}\left(t_{0}\right)\right)\sin\left(\alpha_{T/R}^{\mathrm{LoS}}\left(t_{0}\right)\right)+\int_{t_{0}}^{t}\left(v_{T,R}\left(t\right)\right)\cdot \sin\left(\alpha_{T,R}^{v}\left(t\right)\right)\cos\left(\beta_{T,R}^{v}\left(t\right)\right)dt\right)}^{2}\\ +{\left(D_{\mathrm{LoS}}^{}\left(t_{0}\right)\sin\left(\beta_{T/R}^{\mathrm{LoS}}\left(t_{0}\right)\right)+\int_{t_{0}}^{t}\left(v_{T,R}\left(t\right)\right)\cdot \sin\left(\beta_{T,R}^{v}\left(t\right)\right)dt\right)}^{2} \end{array}}$$

(19) $$D_{T/R,j}^{}\left(t\right)=\sqrt{\begin{array}{c} {\left(D_{T/R,j}^{}\left(t_{0};t\right)\cos\left({\bar{\alpha }}_{T/R}^{j}\left(t_{0}\right)\right)\cos\left({\bar{\beta }}_{T/R}^{j}\left(t_{0}\right)\right)+\int_{t_{0}}^{t}v_{T/R}\left(t\right)\cos\left(\alpha_{T/R}^{v}\left(t\right)\right)\cos\left(\beta_{T/R}^{v}\left(t\right)\right)dt\right)}^{2}\\ +{\left(D_{T/R,j}^{}\left(t_{0};t\right)\cos\left({\bar{\beta }}_{T/R}^{j}\left(t_{0}\right)\right)\sin\left({\bar{\alpha }}_{T/R}^{j}\left(t_{0}\right)\right)+\int_{t_{0}}^{t}v_{T/R}\left(t\right)\sin\left(\alpha_{T/R}^{v}\left(t\right)\right)\cos\left(\beta_{T/R}^{v}\left(t\right)\right)dt\right)}^{2}\\ +{\left(D_{T/R,j}^{}\left(t_{0};t\right)\sin\left({\bar{\beta }}_{T/R}^{j}\left(t_{0}\right)\right)+\int_{t_{0}}^{t}v_{T/R}\left(t\right)\sin\left(\beta_{T/R}^{v}\left(t\right)\right)dt\right)}^{2} \end{array}}$$

where $\alpha_{T/R}^{v}\left(t\right)$ and $\beta_{T/R}^{v}\left(t\right)$ denote the relative travel direction between the UAV and vehicle on the azimuth and elevation plane, respectively.

Based on the geometric relationships, the time-variant angles such as the EAoD, AAoD, EAoA, and AAoA of LoS path under dynamic U2V communication scenarios can be obtained respectively by

(20) $$\alpha_{T/R}^{\mathrm{LoS}}\left(t\right)=\left\{\begin{array}{c} \arccos\left(\frac{∥D_{T/R,\mathrm{LoS}}^{x}\left(t\right)∥}{\sqrt{{∥D_{T/R,\mathrm{LoS}}^{x}\left(t\right)∥}^{2}+{∥D_{T/R,\mathrm{LoS}}^{y}\left(t\right)∥}^{2}}}\right),D_{T/R,\mathrm{LoS}}^{x}\left(t\right)\geq 0\\ \pi -\arccos\left(\frac{∥D_{T/R,\mathrm{LoS}}^{x}\left(t\right)∥}{\sqrt{{∥D_{T/R,\mathrm{LoS}}^{x}\left(t\right)∥}^{2}+{∥D_{T/R,\mathrm{LoS}}^{y}\left(t\right)∥}^{2}}}\right),D_{T/R,\mathrm{LoS}}^{x}\left(t\right)<0 \end{array}\right.$$

(21) $$\beta_{T/R}^{\mathrm{LoS}}\left(t\right)=\arcsin\left(\frac{∥D_{T/R,\mathrm{LoS}}^{z}\left(t\right)∥}{D_{\mathrm{LoS}}^{}\left(t\right)}\right)$$

where $D_{T/R,\mathrm{LoS}}^{x}\left(t\right)$, $D_{T/R,\mathrm{LoS}}^{y}\left(t\right)$, $D_{T/R,\mathrm{LoS}}^{z}\left(t\right)$ denote the *x*, *y*, *z* component of $D_{T/R,\mathrm{LoS}}^{}\left(t\right)$, respectively. Then, the Doppler frequency of LoS path can be rewritten as

(22) $$\begin{array}{cc} f_{}^{\mathrm{LoS}}\left(t\right) & =\frac{v_{T}\left(t\right)\cdot r_{T}^{\mathrm{LoS}}\left(t\right)}{\lambda }+\frac{v_{R}\left(t\right)\cdot r_{R}^{\mathrm{LoS}}\left(t\right)}{\lambda }\\ \; & =\frac{v_{T}\left(t\right)\left(\cos\left(\alpha_{T}^{\mathrm{LoS}}\left(t\right)-\alpha_{T}^{v}\left(t\right)\right)\cos\beta_{T}^{\mathrm{LoS}}\left(t\right)\cos\beta_{T}^{v}\left(t\right)+\sin\beta_{T}^{\mathrm{LoS}}\left(t\right)\sin\beta_{T}^{v}\left(t\right)\right)}{\lambda }\\ \; & \;\;\;\;\;\;+\frac{+v_{R}\left(t\right)\left(\cos\left(\alpha_{R}^{\mathrm{LoS}}\left(t\right)-\alpha_{R}^{v}\left(t\right)\right)\cos\beta_{R}^{\mathrm{LoS}}\left(t\right)\cos\beta_{R}^{v}\left(t\right)+\sin\beta_{R}^{\mathrm{LoS}}\left(t\right)\sin\beta_{R}^{v}\left(t\right)\right)}{\lambda }. \end{array}$$

For the NLoS paths, the mean angles of time-variant EAoD, AAoD or EAoA, AAoA can be calculated respectively by

(23) $${\bar{\alpha }}_{T/R}^{j}\left(t\right)=\left\{\begin{array}{c} \arccos\left(\frac{∥D_{T/R,j}^{x}\left(t\right)∥}{\sqrt{{∥D_{T/R,j}^{x}\left(t\right)∥}^{2}+{∥D_{T/R,j}^{y}\left(t\right)∥}^{2}}}\right),D_{T/R,j}^{x}\left(t\right)\geq 0\\ \pi -\arccos\left(\frac{∥D_{T/R,j}^{x}\left(t\right)∥}{\sqrt{{∥D_{T/R,j}^{x}\left(t\right)∥}^{2}+{∥D_{T/R,j}^{y}\left(t\right)∥}^{2}}}\right),D_{T/R,j}^{x}\left(t\right)<0 \end{array}\right.$$

(24) $${\bar{\beta }}_{T/R}^{j}\left(t\right)=\arcsin\left(\frac{∥D_{T/R,j}^{z}\left(t\right)∥}{D_{T/R,j}^{}\left(t\right)}\right).$$

Similarly, the time-variant delays of LoS and NLoS paths are related with the transmission distance and they can be calculated respectively by

(25) $$\tau_{}^{\mathrm{LoS}}\left(t\right)=\frac{D_{\mathrm{LoS}}^{}\left(t\right)}{c}$$

(26) $${\bar{\tau }}_{}^{j}\left(t\right)=\frac{D_{T,j}^{}\left(t\right)+D_{R,j}^{}\left(t\right)}{c}$$

where *c* is the speed of light.

### 3.2. Data-Based Stochastic Parameters

Note that the channel parameters obtained by the geometric relationships cannot reflect the statistical properties of random rays within the NLoS path. In this paper, we have conducted simulations in 28 GHz based on the RT method for up to 7200 different UAV channels under four scenarios, i.e., urban, forest, hill, and sea. Huge amount of obtained channel data is used to analyze the stochastic characteristic of random rays. Based on these analytical results, the computation method of intra-path or ray parameters are developed as follows.

**Figure 3:**
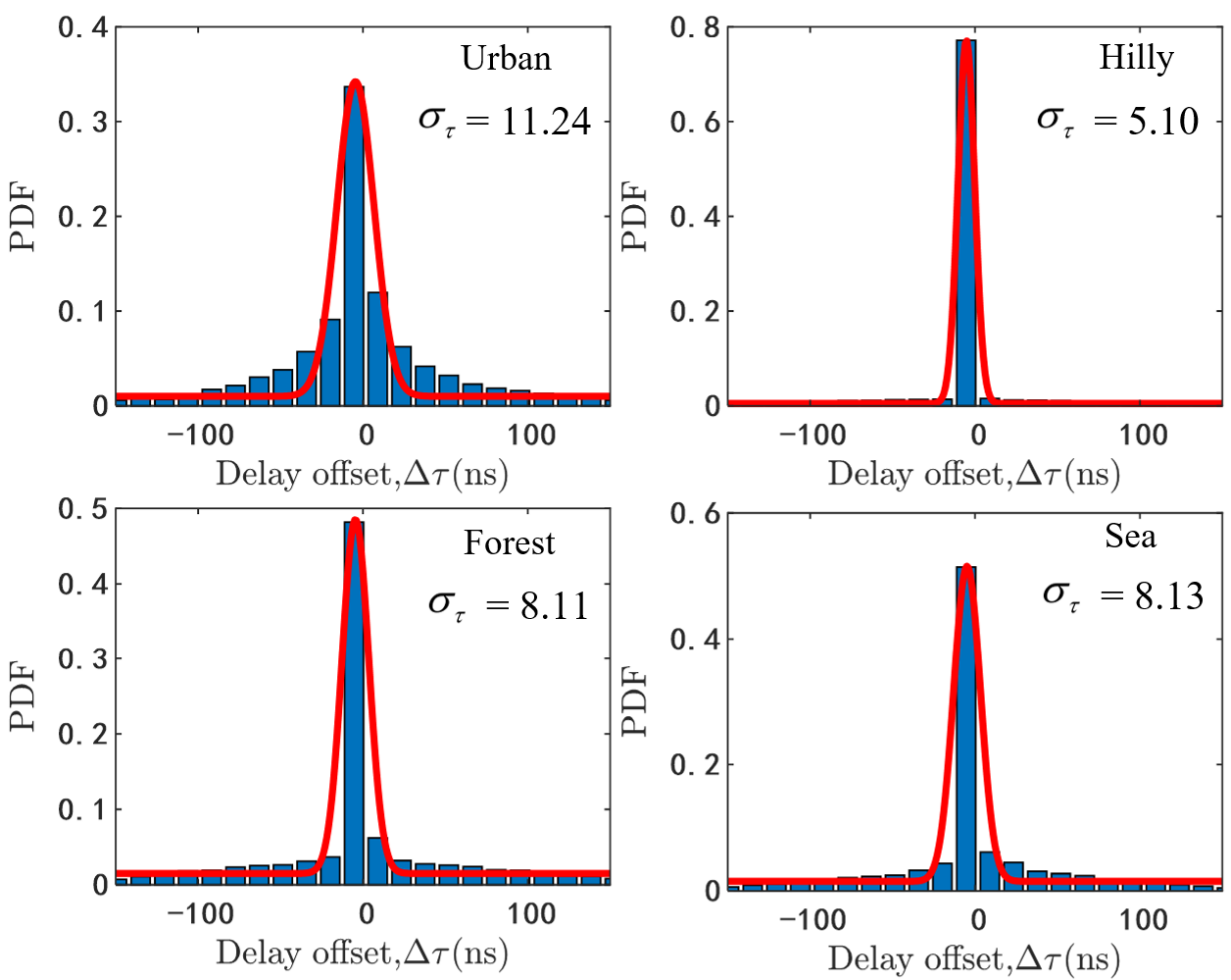
The PDFs of ray delay offset.

Each NLoS path may include several rays with different delays, which can be described as the relative delay between the intra-path ray delay and mean path delay. The data-based analytical results of ray delay offset under different scenarios are shown in Figure 3. As we can see that it is more likely to arise the delay offset under the urban scenario and the value of the ray delay offset with high probability can be up to 10 ns since the urban scenario has rich scatterers. In the other simulation scenarios, the values of the ray delay offset with high probability are normally less than 6 ns.

Moreover, it can be seen from Figure 3 that the PDFs of delay offset fit well with modified Gaussian distribution with zero mean value. Therefore, the delay offset of the *m*th ray within *j*th path can be modeled as

(27) $$f\left(\Delta \tau_{m}^{j}\right)=\frac{a_{\tau }}{\sqrt{2\pi }\sigma_{\tau }}\exp\left(-\frac{{\left(\Delta \tau_{m}^{j}\right)}^{2}}{2{\sigma_{\tau }}^{2}}\right)+b_{\tau }$$

where $a_{\tau }$ and $b_{\tau }$ are the weight factor and correction factor of the magnitude respectively, and $\sigma_{\tau }^{}$ is the standard deviation of delay offset. In Figure 3, $a_{\tau }$ is 9.36, 9.80, 9.56, 10.20, $\sigma_{\tau }^{}$ is 11.24, 5.10, 8.11, 8.13 and $b_{\tau }$ is 0.011, 0.005, 0.015, 0.016 in four different scenarios. Then, the ray delay can be generated by

(28) $$\tau_{m}^{j}\left(t\right)={\bar{\tau }}_{}^{j}\left(t\right)+\Delta \tau_{m}^{j}.$$

Furthermore, the power of each ray can be calculated according to the ray delay by the exponential distribution as

(29) $$P_{m}^{j}\left(t\right)=\xi \exp\left(-1000\xi \tau_{m}^{j}\left(t\right)\right)$$

where ξ is the rate parameter of exponential distribution. According to the analytical results of the simulation data, ξ is well fitted by 5.85, 26.7, 22.8, and 25.05 in four typical scenarios. When the power of LoS path is normalized to be 0 dB, the power of each ray can be normalized as

(30) $${\tilde{P}}_{m}^{j}\left(t\right)=\frac{P_{m}^{j}\left(t\right)}{A\left(\theta^{\mathrm{LoS}},\varphi^{\mathrm{LoS}}\right)\cdot K\left(t\right)\cdot \sum_{m=1}^{M}P_{m}^{j}\left(t\right)}$$

where the total cluster powers should be 1/(K(t)+1) as shown in (2).

**Figure 4:**
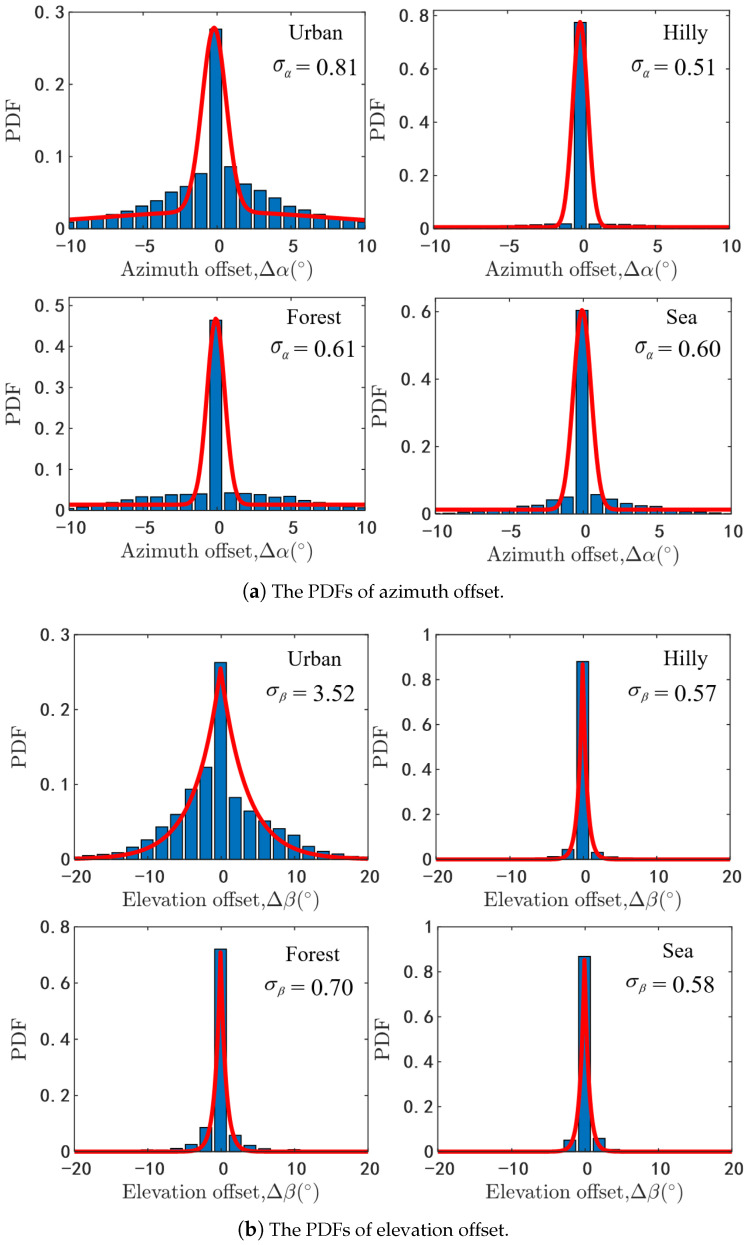
The PDFs of the ray angle offset.

Similarly, the mean angles of the NLoS path are along with ray angle offset as well. The analytical results of the azimuth and elevation angle offset are shown in Figure 4. As can be seen that the values of the azimuth angle offset with high probability are smaller than the ones of elevation angle offset, because the width of the scatterers is normally shorter than the height of the scatterers especially under the urban scenario. Moreover, we can find that the PDFs of the azimuth and elevation angle offset can be fitted well by modified Gaussian distribution and Laplacian distribution respectively, which is similar with the recommendation in [27]. For the azimuth offset of AAoA or AAoD, the PDFs can be described by $a_{\alpha }$, $\sigma_{\alpha }^{}$, and $\Delta \alpha_{m}^{j}$ as

(31) $$f\left(\Delta \alpha_{m}^{j}\right)=\frac{a_{\alpha }}{\sqrt{2\pi }\sigma_{\alpha }}\exp\left(-\frac{{\left(\Delta \alpha_{m}^{j}\right)}^{2}}{2{\sigma_{\alpha }}^{2}}\right)+b_{\alpha }.$$

In Figure 4a, $a_{\alpha }$ is 0.51, 0.98, 0.68, 0.89, $\sigma_{\alpha }^{}$ is 0.81, 0.51, 0.60, 0.60 and $b_{\alpha }$ is 0.017, 0.006, 0.014, 0.013, respectively. The PDFs of elevation offset EAoA or EAoD can be expressed by $\Delta \beta_{m}^{j}$ as

(32) $$f\left(\Delta \beta_{m}^{j}\right)=\frac{a_{\beta }}{2\sigma_{\beta }}\exp\left(-\frac{\left|\Delta \beta_{m}^{j}\right|}{\sigma_{\beta }}\right).$$

In Figure 4b, $a_{\beta }$ is 1.810, 1.008, 1.005, 1.007 and $\sigma_{\beta }$ is 3.52, 0.57, 0.70, 0.58 in different scenarios. Then the ray angle can be generated by

(33) $$\alpha_{T/R,m}^{j}\left(t\right)={\bar{\alpha }}_{T/R}^{j}\left(t\right)+\Delta \alpha_{m}^{j}$$

(34) $$\beta_{T/R,m}^{j}\left(t\right)={\bar{\beta }}_{T/R}^{j}\left(t\right)+\Delta \beta_{m}^{j}.$$

Finally, the Doppler frequency of NLoS path should be calculated by the relative velocity both between the UAV and scatterers and between the vehicle and scatterers. However, the scatterers in this paper are assumed to be static and then the Doppler frequency of NLoS path can be got equivalently by the velocity of the UAV and vehicle. Thus, the Doppler frequency of each ray $f_{m}^{j}\left(t\right)$ within *j*th NLoS path can be described as

(35) $$f_{m}^{j}\left(t\right)=f_{T,m}^{j}\left(t\right)+f_{R,m}^{j}\left(t\right)$$

where $f_{T,m}^{j}\left(t\right)$ and $f_{R,m}^{j}\left(t\right)$ can be further expressed as

(36) $$\begin{array}{cc} f_{T,m}^{j}\left(t\right) & =\frac{v_{T}\left(t\right)\cdot r_{T,m}^{j}\left(t\right)}{\lambda }\\ \; & =\frac{v_{T}\left(t\right)\left(\cos\left(\alpha_{T,m}^{j}\left(t\right)-\alpha_{T}^{v}\left(t\right)\right)\cos\beta_{T,m}^{j}\left(t\right)\cos\beta_{T}^{v}\left(t\right)+\sin\beta_{T,m}^{j}\left(t\right)\sin\beta_{T}^{v}\left(t\right)\right)}{\lambda } \end{array}$$

(37) $$\begin{array}{cc} f_{R,m}^{j}\left(t\right) & =\frac{v_{R}\left(t\right)\cdot r_{R,m}^{j}\left(t\right)}{\lambda }\\ \; & =\frac{v_{R}\left(t\right)\left(\cos\left(\alpha_{R,m}^{j}\left(t\right)-\alpha_{R}^{v}\left(t\right)\right)\cos\beta_{R,m}^{j}\left(t\right)\cos\beta_{R}^{v}\left(t\right)+\sin\beta_{R,m}^{j}\left(t\right)\sin\beta_{R}^{v}\left(t\right)\right)}{\lambda }. \end{array}$$

## 4. Simulation Results and Validations

**Figure 5:**
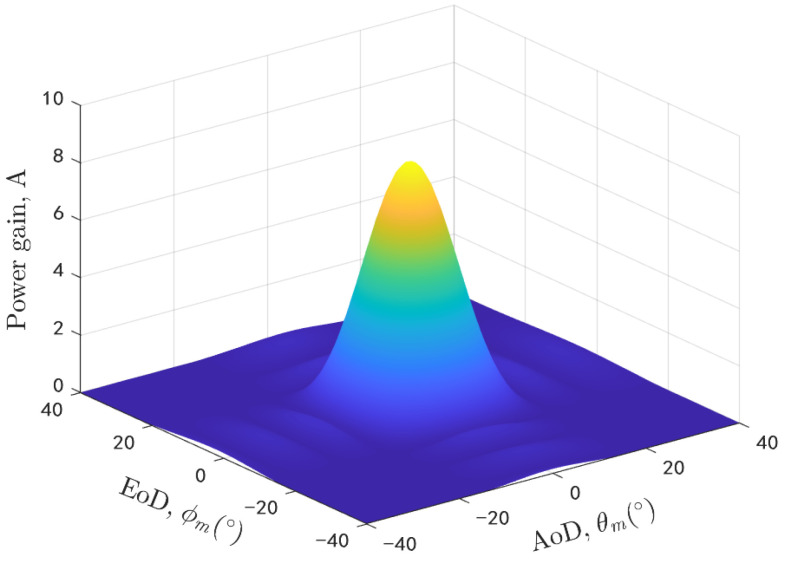
The power gain coefficient of 3D beam in different angles.

To illustrate and verify the proposed U2V beamforming channel model, we take the urban scenario as an example. The 3D trajectories of both terminals and 3D beam are considered. It should be mentioned that the angular error of beam tracking is ignored and the rest simulation parameters are shown in Table 1. Moreover, the generation method of 3D beam in [28] is adopted in this paper and the beam width is assumed to be 22.5 degrees. The power gain coefficient of 3D beam is simulated and given in Figure 5. As we can see that the power gain will decay with the azimuth angle and elevation angle deviating from the beam center.

**Figure 6:**
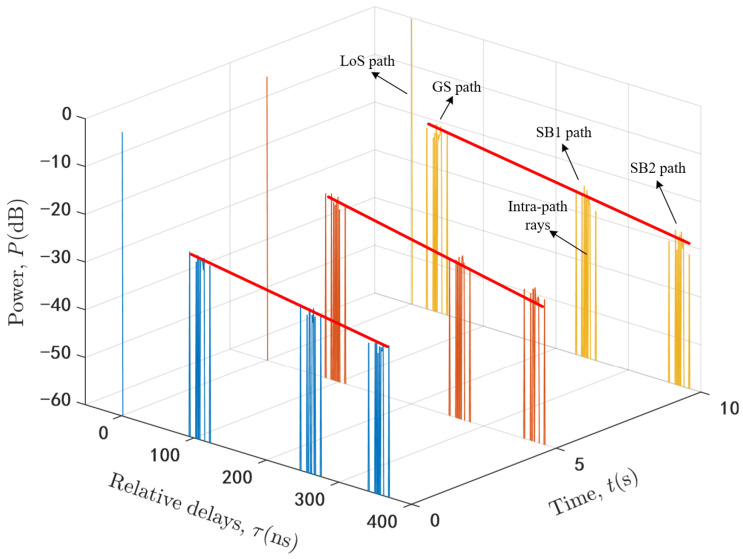
The time-variant normalized PDPs of proposed U2V channel model.

The delay, angle, and power of each path and intra-path ray become complicated due to the movement of terminals and 3D beamforming. Based on the above computation method, we run the proposed channel model and obtain the time-variant normalized PDPs at three different time instants $t_{1}$ = 0 s, $t_{2}$ = 5 s, and $t_{3}$ = 10 s as shown in Figure 6. As we can see, each NLoS path includes several intra-path rays. Although the path power and intra-path ray power will be affected a little by the gain coefficient of 3D beam, the trend of mean path power in dB is linear attenuation. It should be noted that the exponential expression in (29) can be rewritten to a linear expression with respect to the power in dB.

**Figure 7:**
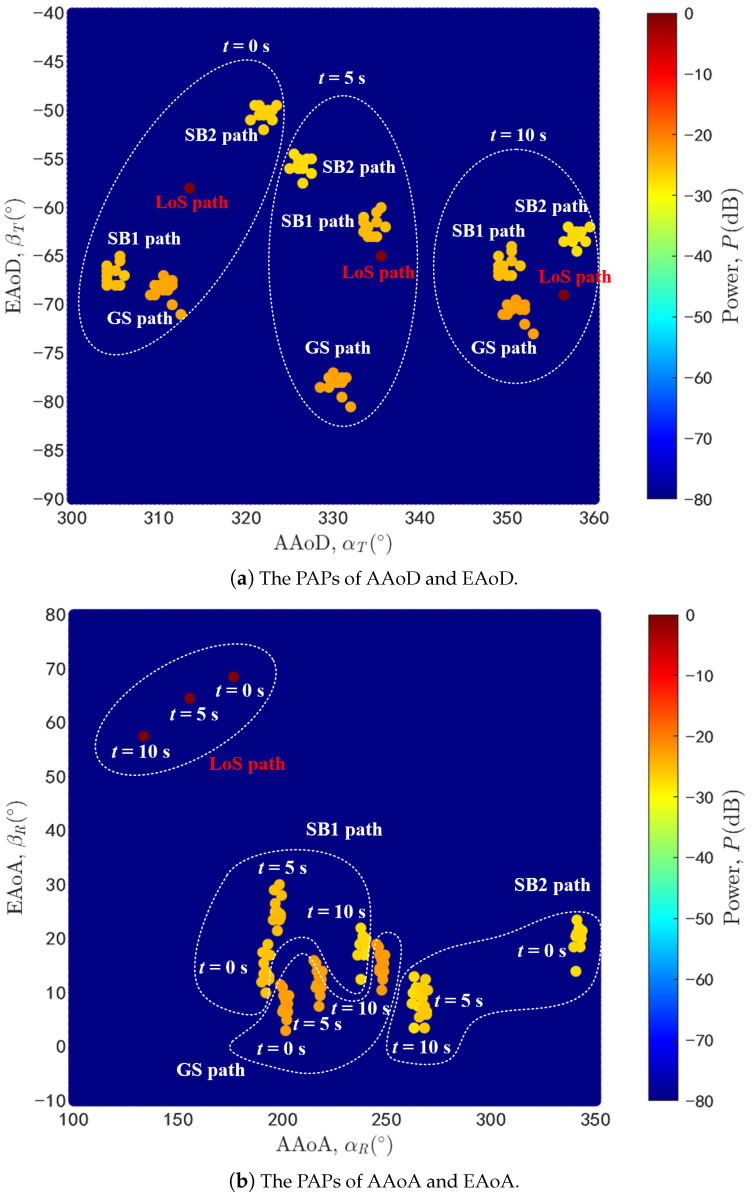
The time-variant PAPs of LoS path and NLoS paths.

Similarly, the angle parameters can be simulated according to the geometric parameters of the simulation scenario and above statistical distribution of the angle offset. The time-variant PAPs of LoS path and rays within three NLoS paths at three different time instants $t_{1}$ = 0 s, $t_{2}$ = 5 s, and $t_{3}$ = 10 s are shown in Figure 7. As we can see from the Figure 7a, taking the AAoD and EAoD of LoS path as the central point, the deviation range of NLoS paths at different time instant are all limited within 22.5 degrees which is consistent with the beam width. In addition, the beam points to different direction at different time instants due to the movement of the UAV and vehicle. In Figure 7b, the spread range of AAoA is up to 200 degrees which is much wider than the EAoA since the scatterers may distribute around the vehicle arbitrary but the height of the scatterers is limited. Moreover, the vehicle antenna is usually close to the ground and thus the EAoA would be within $\left[0^{\circ }{\;}_{}^{}90^{\circ }\right]$.

The ACF is an important second order statistical property which describes the fading correlation over time. It is usually used to verify the correctness of theoretical channel model. The theoretical normalized ACF of proposed model by the definition can be expressed as

(38) $$\begin{array}{cc} r_{hh}\left(\Delta t;t\right) & =\frac{E\left[h^{*}\left(t\right)h\left(t+\Delta t\right)\right]}{\sqrt{E\left[{\left|h^{*}\left(t\right)\right|}^{2}\right]E\left[{\left|h(t+\Delta t)\right|}^{2}\right]}} \end{array}$$

where $E\left(\cdot \right)$ is the expectation function and ${\left(\cdot \right)}^{*}$ is the complex conjugate.

**Figure 8:**
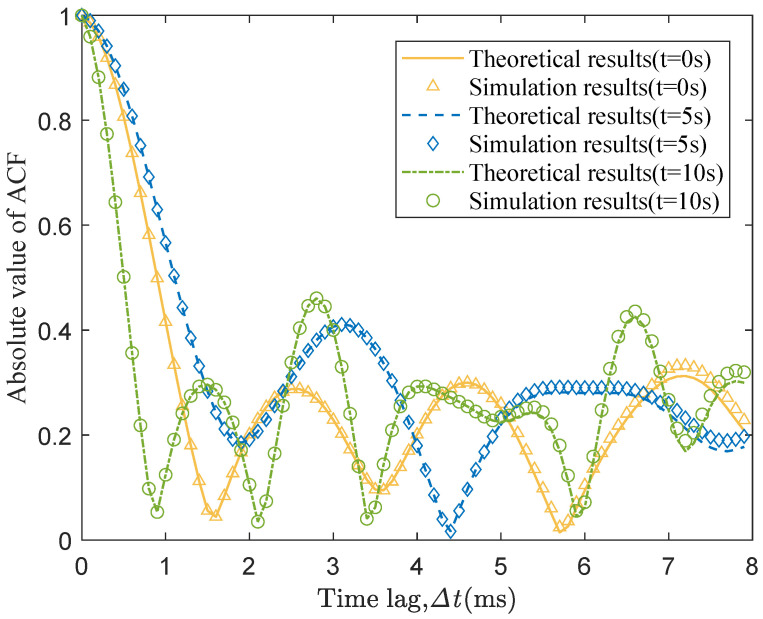
The simulated and theoretical time-variant ACFs at three time instants.

**Figure 9:**
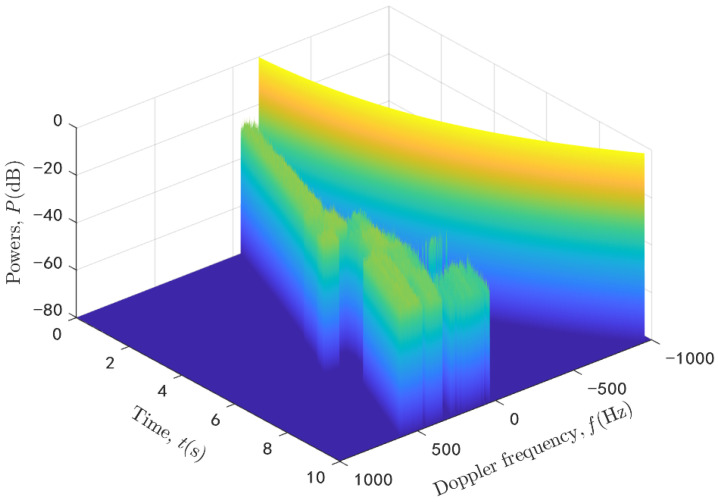
The simulated 3D time-variant DPSDs.

By submitting (2) into (38), we can obtain the theoretical ACF of proposed model. On the other hand, the simulated ACF can be obtained by using the generated channel data. The theoretical and simulated ACFs are compared in Figure 8 at three different time instants $t_{1}$ = 0 s, $t_{2}$ = 5 s, and $t_{3}$ = 10 s. It can be seen that the ACFs change over time due to the time-variant channel parameters and the simulated results fit well with the theoretical ones. Furthermore, we can get the DPSDs by using the Fourier transform of ACFs and the simulated results of the 3D time-variant DPSDs are shown in Figure 9. As was shown, the Doppler frequency changes in a complicated way under the U2V communication scenario due to the 3D trajectory and 3D beamforming. It should be noted that all the LoS path and NLoS paths are considered together in the simulation results of the ACFs and DPSDs.

**Figure 10:**
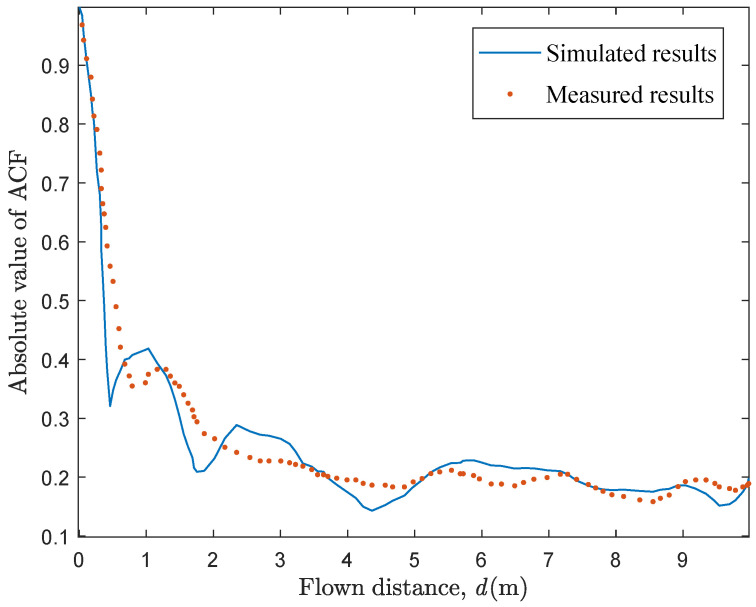
The simulated and measured results of ACFs.

To further verify the consistency of proposed channel model with realistic channels, the simulated ACF is compared with the measured one in [29]. It should be mentioned that there is very little literature involving UAV mmWave channel measurement [30,31,32] and none of them so far analyzed the measured ACFs. Noting that GBSM is a general channel modeling method which can be applied in both the sub-mmWave band and mmWave band, the proposed model could be used for the sub-mmWave GBSM by adjusting some channel parameters. Thus, the measured ACF of sub-mmWave channel in [29] is chosen. Here, we ignore the effect of beam width and reconfigure some parameters as $f_{0}$ = 2 GHz, $∥L_{\mathrm{tx}}\left(t_{0}\right)∥$ = 300 m, *K* = 7.4 dB and $∥v_{\mathrm{rx}}\left(t\right)∥$ = 1.2 m/s according to the measurement condition. Figure 10 gives the comparison between the simulated and measured results and the well agreement reflects the generality and correctness of the proposed model.

## 5. Conclusions

This paper proposed a 3D non-stationary GBSM for U2V mmWave beam channel by considering the 3D arbitrary trajectory of both terminals, the rotation of 3D antenna array, and 3D beam-forming. To achieve the tradeoff of complexity and accuracy, the model only includes the LoS path and three strongest NLoS paths. Moreover, the hybrid computation method of channel parameters has also been given, which is divided into a geometry-based part and a data-based stochastic part to guarantee both the precision and efficiency. At last, the simulation results of PDPs, ACFs, and DPSDs have been compared with the theoretical and measured ones. In the future, we will perform more channel measurements to verify the channel model as well as optimize the calculation method of channel parameters.

## Figures and Tables

**Table 1:** Simulation parameters.

Definition	Value	Definition	Value
$v_{T}\left(t\right)$	10+0.5t m/s	$v_{R}\left(t\right)$	2+t m/s
$\alpha_{T}^{v}\left(t\right)$	$120\;-\;2t^{\circ }$	$\beta_{T}^{v}\left(t\right)$	$6+5t^{\circ }$
$\alpha_{R}^{v}\left(t\right)$	$-120+2t^{\circ }$	$\beta_{R}^{v}\left(t\right)$	$0^{\circ }$
λ	3/280 m	*K*	7 dB
$∥L_{T}\left(t_{0}\right)∥$	400 m	$∥L_{R}\left(t_{0}\right)∥$	100 m
$\sigma_{\tau }^{}$	11.24	$\sigma_{\alpha }^{}$	0.81
$\sigma_{\beta }^{}$	3.52	*M*	12
